# Activity Inference for Ambient Intelligence Through Handling Artifacts in a Healthcare Environment

**DOI:** 10.3390/s120101072

**Published:** 2012-01-20

**Authors:** Francisco E. Martínez-Pérez, Jose Ángel González-Fraga, Juan C. Cuevas-Tello, Marcela D. Rodríguez

**Affiliations:** 1 Facultad de Ingeniería, Universidad Autónoma de Baja California, Km 103 Carretera Tijuana-Ensenada, Ensenada, B.C. 022860, México; 2 Facultad de Ciencias, Universidad Autónoma de Baja California, Km 103 Carretera Tijuana-Ensenada, Ensenada, B.C. 022860, México; E-Mail: angel_fraga@uabc.edu.mx; 3 Facultad de Ingeniería, Universidad Autónoma de San Luis Potosí, Av. Manuel Nava No. 8, Zona Universitaria, San Luis Potosí, S.L.P. 78290, México; E-Mail: cuevas@uaslp.mx; 4 Facultad de Ingeniería, Universidad Autónoma de Baja California, Mexicali, B.C. 21280, México; E-Mail: marcerod@uabc.edu.mx

**Keywords:** activity inference, activity representation, artifact recognition, ambient intelligence

## Abstract

Human activity inference is not a simple process due to distinct ways of performing it. Our proposal presents the SCAN framework for activity inference. SCAN is divided into three modules: (1) artifact recognition, (2) activity inference, and (3) activity representation, integrating three important elements of Ambient Intelligence (AmI) (artifact-behavior modeling, event interpretation and context extraction). The framework extends the roaming beat (RB) concept by obtaining the representation using three kinds of technologies for activity inference. The RB is based on both analysis and recognition from artifact behavior for activity inference. A practical case is shown in a nursing home where a system affording 91.35% effectiveness was implemented *in situ*. Three examples are shown using RB representation for activity representation. Framework description, RB description and CALog system overcome distinct problems such as the feasibility to implement AmI systems, and to show the feasibility for accomplishing the challenges related to activity recognition based on artifact recognition. We discuss how the use of RBs might positively impact the problems faced by designers and developers for recovering information in an easier manner and thus they can develop tools focused on the user.

## 1. Introduction

The purpose of Ambient Intelligence (AmI) is to integrate multiple sensors within a distributed interface, focused on the user, and includes research and development of algorithms based on data acquisition and collaborative processing, data fusion, event interpretation, context extraction and behavior modeling in an environment [1]. Nowadays AmI systems have been deployed in several applications in environments such as homes, factories, offices, meeting rooms, classrooms, vehicles, and museums; hospitals and nursing homes are not the exception. Typically several types of sensors are embedded such as microphones, video cameras, movement sensors, radio frequency identification (RFID) and others to sense human activity [2–4]. The main objective of these devices is to gather information from a scenario in order to obtain an interpretation from the environment surrounding the user, so that with this information the activities that a user undertakes are known, thus making it possible to provide information services (see [5]). In other words, we are interested in knowing what activities humans undertake, and the challenge is to recognize activities in complex situations [6]. Commonly, human activities are performed not only in a sequential way, but also in an interleaved and concurrent manner [6,7]. Moreover, the quantity of sensors required to recognize human activities is numerous.

In this paper, we propose a framework to infer human activities based on handling artifacts, and we use the term artifact to refer to items that are labeled or have an electronic device embedded that gathers information automatically. Our framework is divided in three modules: (1) the first module refers to artifact recognition, which focuses on both data acquisition and conversion from three types of technology; (2) the second module uses the data conversion with the aim of activity inference using the roaming beat concept, which is discussed below; (3) the third module focuses on activity representation based on beats produced by artifacts handled in the activities. These three modules allow us to integrate three important elements of AmI for activity inference: artifact-behavior modeling, event interpretation and the context extraction. Thus, these three modules are the basis of our AmI system in several scenarios of the healthcare environment for elderly people, including capture, inference and visualization of activities. In this paper, we present a practical case of a nursing home, where we recognize artifacts by using three technologies: RFID, accelerometers, and computer vision.

Integral to this framework, we show how our concept of the roaming beat (RB) [8] is obtained from distinct technologies within a healthcare environment, where the RB represents a set of images in a time span obtained by artifact-behavior when an activity is performed; it allows us to recover several kinds of context from the setting such as identity, location, time and activity based on the analysis of artifact behavior, and the RB concept allows activity representation to be useful for:

- Incorporating several technologies for capturing the artifacts behavior.
- Representing and understanding artifacts behavior to perform an activity.
- Activity inference based on the artifacts manipulation, and solving the challenge of activity recognition.
- Implementing systems that allow the activity monitoring and information recovery of a scenario via images or video segments.

The framework demonstrated here allows designers and developers to perform activity inference in an easier way than has previously been reported, and allows them to produce tools that might provide ambient intelligence in nursing homes. This paper is organized as follows: Section 2 presents the related work to this research. Section 3 introduces our framework for activity inference. In Section 4, we present our practical case under study. Our discussion is in Section 5, and finally Section 6 provides the conclusions.

## 2. Related Work

As mentioned above, AmI refers to the integration of multiple sensors within a distributed interface to assist persons in their daily activities. To achieve this, it is necessary to develop a detailed analysis of each scenario where one wants to integrate AmI. Most of the research has been focused on the analysis and design of systems to assist nurses in an automatic way by using technology [9]. The central part of these investigations is data gathering because it is important for identifying needs and establishing requirements. When obtaining the requirements, one needs to gather sufficient, accurate and relevant data, so a set of stable requirements can be produced. One technique to obtain data is observation, in particular ethnography [10], and it is supported by existing frameworks for activity inference or new ones that include simple questions like who? where? what? and why?. However, sufficient questions are required to help designers and developers to understand the user context, activities, and objectives of specific scenarios, so the implementation of sensors that would allow for analysis of human behavior in an automatic way [11,12].

In healthcare environments some researchers have explored problems related to the study of human behavior by using several types of sensors. We grouped in two classes these investigations:

1. Research focused on generating tools for monitoring patients and gathering behavioral patterns on people; and
2. Research for human activities recognition.

### 2.1. Tools for Monitoring Patients and Gathering Behavioral Patterns on People

Most of the research in nursing homes is devoted to the development of tools that are to be used by nurses for monitoring patients [9]. The monitoring is carried out by capturing information from several types of sensors that obtain a complete perspective about the health status of an elderly person. This provides the patterns of behavior and the execution of a patient’s Activities of Daily Living (ADL). For example, a monitoring system has been developed with a bio-sensor for monitoring the physiological condition of an elderly person and an RFID sensor to know his localization; both sensors are carried by the elderly person. The information from these sensors is used by the nursing home to detect a patient’s emergency or accident [13]. A similar research study employs RFID and WiFi plus intelligent agents to send alerts notifying a falling down or other health risks through messages to mobile devices [2]. However in this last study, the event context is omitted, before, during and after the identified event, and consequently the cause is unknown. Other researchers use video images, so they can record events. They can track an object in a scene by using computer vision techniques, and they start recording when the object appears until the object disappears from the scene. This allows them to detect the cause of an accident [3]. All these studies have focused on elderly people with mobility or independence, and they do not take in account elders with restricted mobility (ERM), also these studies neither record the activities of the caregivers in nursing homes nor evaluate the quality of care. This environment is especially important because ERM are dependent: an accident or chronic disease pushes them to stay at the same place for long periods of time, so they depend on caregivers to survive. Thus, the quality of life of ERM depends on the caregiver in these nursing homes. Consequently, recording the activities of the caregiver is important to have the full context of the scene.

However, it is necessary to perform an analysis of the environment within the nursing home to achieve full monitoring of elderly people (independent or not) [9,10]. Some researchers present tools for communication among caregivers or nurses based on ethnographic studies [10], where the requirements and implications are the developing of an application for communicating among caregivers. However, this application only focuses on independent elderly people [9].

Some research has been done on dependent elderly people, or immobile patients. That research is mainly for monitoring bed surface temperature automatically by sensors and activating a fan via a microcontroller to make decisions (hotter/cooler); this is to avoid bed sores on the patient’s skin [14].

### 2.2. Approaches for Activity Recognition

A number of projects have studied algorithms, methods and devices for the automatic recognition of user’s activities. Recognition of human activities also enables the real-time monitoring of patients, children, and elderly persons. Some research has been focused on recognition of human activities using pattern recognition, computer vision or other kinds of technologies such as RFIDs, biosensors and accelerometers and they focused on healthcare environment where the major requirement of the health care system is reducing risks to people [15]. For example, the ADL of elderly people such as preparing drinks or taking medicine have been inferred with 73% precision by monitoring the objects’ interaction [7]. This was achieved thanks to a glove with RFID to read tags on objects of interest. Other research proposes a rule-based logic programming software for detecting human activities [16]. Along with the rule-based system, some artifacts and RFID tags are used to active the rules. However, the knowledge base requires some time to build and then it must be validated with a human expert who knows the environment perfectly. The precision of this system is not reported, and it is difficult to obtain consistent rules. Other approaches to human activities recognition are implemented by accelerometers and they focus on assessing changes in physical and behavioral profiles. In these approaches the accelerometers have been used in wearable devices for activity classification, and the positioning of the sensors and the selection of relevant features for different activity groups still pose significant research challenges [15,17–19]. Another author has presented an approach where human interaction environments must be understood as any place where people carry out their daily life. They present a solution for the development of systems that require the integration and interoperation of devices and technologies as mentioned above [20]. Research in this area includes hidden Markov models (HMM), which are trained to map the context information of activities carried on by workers within an hospital, e.g., the artifact had been used and associated to somebody else [21]. The context variables, within an activity transition matrix, and a priority vector are the inputs to the model. The activities to be inferred are the hidden states. Inferring this information implies knowing the model that describes the time used by the hospital workers during their activities, per day and per event. This research reports accuracy of 97.6% for activity recognition [21].

In the above research, we see that tag sensing was sequential and avoids the sensing of concurrent activities or linked ones [7]. Some research takes into account this problem and employs techniques such as emerging patterns and HMM [6,22]. The problem is that these approaches require a huge amount of data for training and activity recognition in order to consider the different ways in which an activity can be performed.

Our framework overcomes these difficulties by doing an analysis of the scenario through validating some criteria related to artifact behavior for activity inference. We propose a framework with three modules without the need to attach an artifact to the caregiver; instead we install sensor devices in the environment. The RB concept that we propose was obtained from the analysis of a scenario and contributes to the activity inference based on the artifacts behavior. There is no need for training since the criteria emerges from the analysis taking in account the challenges within the activities recognition.

## 3. SCAN Framework: A Base for Activity Inference

This section introduces our framework named eStimating aCtivities based on Artifact’s behavior aNalysis (SCAN), for developing systems for activity inference. This framework employs the concept of RB for the behavior analysis of physical artifacts for activity inference [8]. This framework can be used as a comprehensive solution for the full process of activity inference by using different technologies, which is helpful for environments with AmI.

The proposed framework is based on the idea that it is possible to classify the activities from the artifact identification used to perform each activity, and by using common sense. For instance, if we detect that a person is using the device to measure blood pressure, and it is possible to identify the interactions and the artifact, then common sense indicates that this person is measuring blood pressure. However, common sense is not enough in some situations to infer an activity, so it is necessary to validate the behavior by some criteria as will be discussed below.

**Figure 1.**
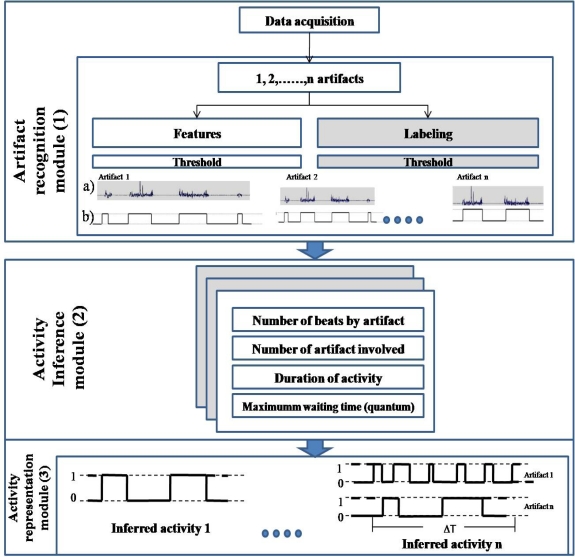
SCAN framework.

Figure 1 shows an example in a framework of the RB concept introduced in [8], which infers the activities based on the artifact’s behavior analysis. SCAN extends the RB concept by obtaining the representation using several technologies for activity inference. The following subsections explain in detail the components within Figure 1. Some authors use activity theory to analyze the artifacts that have been used when a specific activity is performed [23]. In this analysis, the activities are mediated by one or more artifacts. This mediation is essential to the ways in which we can understand artifacts within each activity, and thus allowing for proper activity inference.

The artifacts play an important role for activity inference, because the artifacts are a trigger within an event in the setting. So, RB is a concept for analyzing the artifact role within activity similar to [23]. Based on the artifact analysis, the framework is divided into three modules: (1) artifact recognition; (2) activity inference performed through the analysis of artifact behavior, using roaming beat concept; and (3) activity representation as shown in Figure 1.

### 3.1. Artifact Recognition Module

The purpose of this module is data acquisition, *i.e.*, capturing the artifact recognition to know its behavior and also data conversion (discretization). Because we are interested only in recognizing the artifacts handled and recording the time when this happens in some specific place, the conversion gives us enough information of the artifact behavior. In the proposed approach the accuracy is not affected by the process of inference.

In this study, we use three technologies for artifact recognition: RFID, accelerometer and video cameras, although others could be used. The output from artifact’s recognition is converted into a train of pulses to facilitate understanding and the analysis when the artifacts are handled once an activity is carried on. A conversion process is obtained through discriminating the artifact recognition results by a threshold. The conversion is as follows: a 1 is obtained when an artifact is recognized on the base location and it remains there; a 0 is obtained when the artifact leaves from the base location, so by definition it is being handled. In this way, artifact behavior is modeled based on a concrete meaning of handling, using the technologies aforementioned.

The process is operationalized as shown in Artifact Recognition module of Figure 1(a,b). We show data acquisition from an accelerometer, based on its account tags or features recognition in Figure 1(a). These data are converted as shown in Figure 1(b), so that when an artifact remains immobile in a specific place, its state is 1; and 0 when the artifact is used in the setting. The data acquisition is obtained in a concurrent way and the train of pulses of each artifact is used as input in the activity inference module.

Deciding the kind of technologies for data acquisition that can be used in an application depends on both the user requirements and the kind of context related to the setting desired. All technologies that will be addressed in this work have their advantages and disadvantages, however our objective here precludes discussing them in detail. Rather, we focus on obtaining a similar event interpretation related to activity performed using three kinds of technologies based on artifact behavior, which is described in Section 4.

### 3.2. Activity Inference Module

The purpose of this module is event interpretation, which is produced by the results from the artifact recognition module, with the aim of obtaining the activity inference based on handling artifacts. To accomplish this, the RB concept is used. This concept was obtained through data analysis in a case study in a nursing home and is defined as follows:

“The ability of an artifact to give a time stamp (hour and date) to change from motionless to a mobile state and change from the base location to any other”

[8].

**Figure 2.**
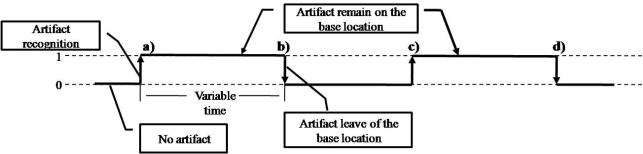
Artifact’s behavior representation (or roaming beat).

This concept describes the artifact behavior when an activity is performed. The behavior is obtained as a result of data conversion when the artifacts are recognized as shown in Figure 2. The behavior analysis is performed as follows:

- The artifact is placed on the base location where it is recognized.
- The artifact remains in the base location for a variable time.
- The artifact leaves from the base location when it will be used for a variable time
- The artifact again appears on the base location and remains for a variable time on it.
- The artifact is removed from the base location.

Taking into account this behavior, the artifact presents just two states, immobile (at rest) and mobile (in use). The immobile state is when an artifact is recognized on the base location and remains at rest. When the artifact is in this state, the user/caregiver performs a preliminary or supplementary action related to the activity such as preparing other artifacts or recording an activity. The mobile state happens when an artifact leaves the base location, and it is supposed that the artifact is being used in performing an activity. These two states are checked by inference rules (or criteria) as mentioned below.

Each artifact produces its own roaming beat (RB) when it is handled. The change of state is called the beat, so several changes of state can be observed as behavior of the artifact (see a, b, c, d in Figure 2) as a result of the artifact recognition in a base location in a time span.

### 3.3. Activity Inference Process

Activity inference process is made through the analysis of artifact-behavior recognition. Therefore, one or more artifacts can be associated with an activity and each artifact follows its own RB. One artifact can appear in more than one activity, however the RB changes according the activity. So, recognizing artifacts play an important role in activity inference. Activity inference is accomplished through the results of the artifact recognition in a time span which produces a train of pulses that belongs to the behavior of the activity. Therefore, it is necessary to analyze each activity behavior to validate its inference through the following criteria:

1. Identifying the number of artifacts involved in an activity.
2. Analyzing the roaming beat for each artifact, by counting at least the minimum number of beats related to the specific activity.
3. Comparing the time span in which the activity was performed (waiting_interval in algorithm).
4. Comparing a quantum of time to a time span produced by the last beat since it occurred. The quantum is obtained by the minimum time in activity duration.

In some cases, activities might arise in which it is necessary to perform a strict analysis (strict case) of the behavior of all artifacts involved to infer the activity. Other cases are more flexible (flexible case) and it is enough to obtain at least two artifact behaviors for inferring it, depending on the result of the activity characterization in a real scenario.

**Figure 3.**
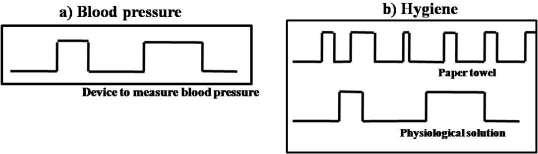
Three roaming beats representations (**a**) Roaming beat related to blood pressure activity; (**b**) Roaming beat related to hygiene activity.

To clarify the criteria, we present the roaming beat analysis of two activities shown in Figure 3. The first one is linked to one artifact (simple activity) and the second is linked to two artifacts (composite activity).

The criteria of the simple activity showed in Figure 3(a) are: (1) the number of artifacts involved is 1; (2) the number of beats established is 4; (3) the time span in which the activity is performed is from 5 to 10 minutes and (4) the maximum waiting time of the last beat is 5 minutes, and that is equal to the minimum time of the time span of the activity. The activity process is as follows: The blood pressure activity is related to only one artifact that is the device to measure blood pressure, when the artifact is recognized on the base location by first time, a beat is created and it is a mark that starts counting the beats produced by artifact recognition. To infer the activity, it is necessary to recognize 4 beats in a time span of activity performed within 5 to 10 minutes. When the last beat is created (4th beat), then the maximum time of waiting for beats starts to count until its threshold of 5 minutes is accomplished. Once that the 5 minutes have finished and another beat has not been recognized, the activity is inferred. The process is repeated for each artifact in a concurrent way and each artifact has its own criteria.

The criteria of the composite activity shown in Figure 3(b) are: (1) the number of artifacts involved is 2; (2) the number of beats of each artifact that must occur to infer the activity is: paper towel ≥6 and physiological solution ≥3; (3) time span in which the activity is performed is from 10 to 30 minutes; and (4) the maximum waiting time of the last beat is 10 minutes, and that is equal to the minimum time of the time span of the activity. The activity process is performed as in the previous example, but in this case two processes are implemented; the number of processes corresponds to the number of artifacts involved.

**Figure 4.**
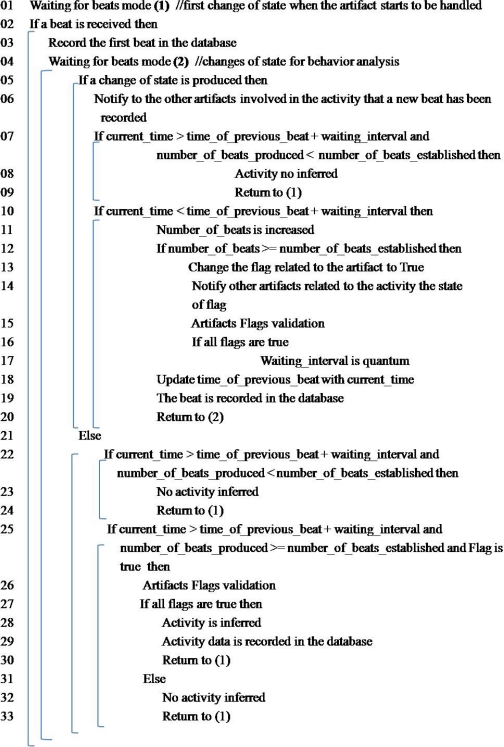
Algorithm for analysis of artifact behavior.

In the examples aforementioned, we must perform three processes and each one must follow the algorithm shown in Figure 4 in a concurrent way.

For implementing the algorithm for analysis of artifact behavior, we use a parent process which enables a child process for each artifact involved in the activity. The parent process manages the artifact’s flags and must perform the communication and coordination between artifacts processes.

The algorithm for analysis of artifact behavior receives as input parameter the signal produced by the artifact recognition module. This algorithm is executed in concurrent way by each artifact process. The artifact’s flag is updated once the number of beats established is accomplished. Also each artifact must load the specifications based on the four criteria aforementioned, *i.e.*, the number of artifacts involved in an activity, the number of beats established, the waiting interval and the quantum.

In this algorithm there are two loops labeled *waiting for beats mode* (line 01 and 04). The first one (line 01) is the waiting process for starting the activity, so the first beat of any artifact related to the activity is verified. So, when a change of state happens, continuing to the next line until the second loop. The second loop in line 04 covers lines 05 to 33. This loop is waiting for the input beats and through a conditional validates if a beat has been received (line 05). If it has not been received then the current time is compared (line 22) with the time related to the last beat produced plus the waiting interval. That is, the activity has an interval of execution, which is given by observing the activity (minimum and maximum time in which the activity is performed). If the conditional in line 22 is true then the whole activity was not performed and the artifact was just put on the base location one time and it remains immobile.

The next time that the artifact starts to move, its change of state will be from 1 to 0. Also, there is another conditional in line 25 whose purpose is to infer the activity. This conditional happens when the artifact behavior has been accomplished and the artifact remains on the base location or disappears from it, so the algorithm is waiting for the quantum to finish (loaded in line 17). Also this conditional must validate three facts (1) the current time is greater than the quantum, (2) the number of beats by artifact has been accomplished and (3) all artifacts flags involved in the activity are true; all of this takes into account the strict or flexible cases of the behavioral artifacts analysis. When these three facts are accomplished, it is possible to infer the activity and then proceed to record the activity and beats in the database. It is important to mention that the last artifact that has produced the last beat of the activity can infer the activity if and only if the three conditions are accomplished (line 25). So, any involved artifact can infer the activity. Therefore, the produced outputs by this module are both beats and activity recorded in the database.

The purpose of the second loop is to capture the artifact behavior. If a change of state is produced, two conditionals (line 07 and 10) are considered. The first one in line 07 validates the time between input beat and the previous beat, also the number of beats that have been recognized. So that if the current time is greater than the time of the previous beat plus waiting interval and the number of beats is not accomplished, then no activity is inferred. In this case, as the activity has started, the waiting interval is loaded with the higher time related to the activity. This case is created when the artifact is removed from the base location and is put somewhere else, but the activity was not performed completely. As consequence the number of beats was less than the number of beats established.

The second conditional (line 10) also validates the artifact behavior when the activity is been performed. So the beats are recognized within waiting interval. In this conditional, the beats are counted, the time related to the previous beat is updated with the current time and the beat is recorded in the database. In this conditional the number of beats is compared with the number of beats established by the artifact. If this number of beats is greater or equal, then the artifact flag must be changed to true and notify this event to the rest of the artifacts involved. When it happens, the quantum value is equal to minimum time related to the activity interval and the waiting interval is equal to quantum (line 17).

When two or more activities share an artifact, the number of beats and the association among artifacts and activities gives the difference. That is, each artifact follows a different behavior depending on the activity on execution, and the artifact is grouped to the other artifacts for inferring the same activity.

Artifact recognition is an important part, because it is based on the behavior that each artifact produces. Each behavior is validated according to the criteria of inference. Therefore, each behavior must be both captured and analyzed in a process. Also, each process must be coordinated with others so that, whether the activity is related to two or more artifacts, there must be an association between artifacts and activities. With this process, we can accomplish the activity recognition in a simple, interleaved and concurrent way.

### 3.4. Activity Representation Module

The objective of this module is to recover the information of activity inference for context retrieval in the setting. This process is accomplished through recovering the beats produced by each artifact when the activity is performed. For instance, Figure 3(a) shows four beats that can be linked to indexes of video sequence captured when the activity of taking blood pressure was performed. So according to the relationship we use these indexes to obtain images from video sequence and prepare the images for activity representation. In the next section, we present two examples where the association of beats and indexes can be seen.

At this point, we have shown the SCAN framework that includes its three modules, and takes into account the RB concept for the first time. The first module presents both artifact recognition and conversion. The conversion allows us to give a simple understanding to the data input of several technologies when the artifacts are handled. The results of this conversion are used as input in the activity inference module, where the artifact behavior is analyzed with the aim of validating the activity inference. It is implemented using four criteria that were obtained through activity characterization of the specific setting. Finally, the activity representation module was presented and it uses the activity inference to show a representation. This last module recovers each beat produced when the activity was performed. Therefore, we believe that it is possible to generalize our application in other kind of scenarios with similar features of performance. To demonstrate generalizability, an example using our framework is implemented in the next section.

## 4. A Practical Case

A practical case is presented in this section as a result of the case study made and presented in [8]. The purpose of the case study was to characterize activities that caregivers performed in providing healthcare of elders with restricted mobility (ERM) in a nursing home. The study was conducted in a private institution in the city of Ensenada, México. This institution mainly focuses on the care of elders with Alzheimer’s disease and senile dementia and it provides daycare services for elders who are recovering from surgery or suffering a terminal disease; in other words, they are elders that depend on caregivers for survival.

The case study was conducted for a period of four months where two patients were observed. These patients live in the nursing home and have restricted mobility. Their caregivers were shadowed for three complete working shifts and interviewed by the researchers. To analyze the data collected, we used grounded theory techniques [24].

As a result of the data, we obtained the activity characterization where it was possible to observe how the roaming beat concept is a fundamental part of the activity inference. We demonstrate how the roaming beat (Figure 2) can be obtained by several technologies. Integrating several technologies is important to complement the information for those cases where missing information is produced by occlusion or a poor signal received by the sensors. The challenge, however, is to have a similar activity interpretation related to the activity performed based on artifacts handling.

Therefore, we present a simple activity as an example using three kinds of technology: RFID, accelerometers and video cameras. This latest technology uses composite correlation filters methods for artifact recognition. The activity performed is taking blood pressure and the artifact used is the device to measure blood pressure.

### 4.1. Setting

**Figure 5.**
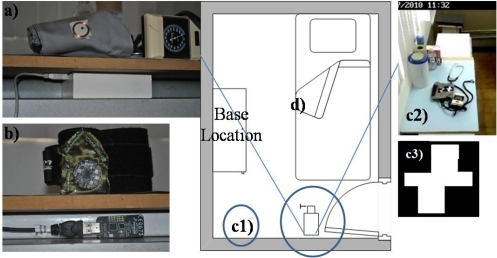
(**a**) RFID tag and antenna; **(b)** Accelerometer device and antenna; (**c**) Cameras installed and label associated to the artifactp; (**d**) Setting lab.

The blood pressure activity was performed in our usability lab. In the setting there was a tool table as a base location where the artifacts are recognized according our RB concept. For the RFID and accelerometer cases, an antenna was installed under the base location as depicted in Figure 5(a,b). For the video camera case, a camera was installed close to the base location approximately two meters away (Figure 5(c1)). In the three cases an extra camera was installed in the ceiling of the room, whose view allowed the observation of the entire setting as shown in Figure 5(d).

#### 4.1.1. Results of Using RFID Sensors

**Figure 6.**
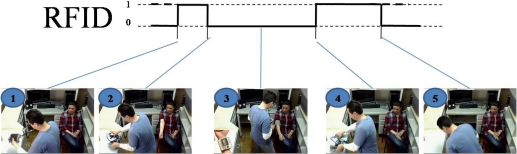
RFID results.

Currently, RFID technology has gained a lot of attention because its low cost, general availability, and location sensing. Its functionality shows good results in artifact recognition and people identification. RFID technology uses two classes for tag recognition called TagGain and TagLoss. The first one is used when the tag is in range of the antenna, so it is recognized. The TagLoss class is activated when the tag is out of range of the antenna. Hence, it is possible to take these values as Booleans where 1 is when the tag is recognized (TagGain) and 0 when not. This interpretation allowed us to obtain the representation shown in Figure 6 using RFID technology, and this representation is similar to the representation presented in Figure 2.

In this example we use a 1023 Phidget RFID model, the tags were passive, so that they have to operate directly from the RF output of the RFID reader and the tags can be read from 3 inches away from the reader. In our case it was enough to put only one antenna, but additional antennas could be necessary for covering the whole base location and recognizing multiple artifacts.

Figure 6 presents a sequence of images obtained by the camera installed in the ceiling of the room, where each one of the steps of the activity performed can be seen. Image 1 in Figure 6 illustrates the moment in which the tag is getting closer to the antenna on the base location. Namely, the caregiver puts the artifact on the tools table. Image 2 shows the caregiver taking the artifact, and it is out of scope of the antenna. Image 3 was recovered from average time within activity performed. Notice that this image is not recovered by a change of state, but is shown to observe the activity that is being performed by the caregiver using the artifact. Image 4 shows the instant which the caregiver again puts the artifact on the base location and it is recognized by the antenna. Finally, image 5 shows the moment that the caregiver takes and puts away the device that measures blood pressure, and it remains out of scope of the antenna.

This is an example using RFID technology linked to the artifacts, and it has other demonstrated functionalities [25]. In this reference, the identification and localization of people is obtained. Another example reported people using a bracelet that is sending the data related to the artifacts handled and detected by the antenna [7]. These data are used to perform the activity inference in a sequential manner. In our scenario, we used the RFID for people identification through linking a tag to the caregiver and installing an antenna in the door of the room, avoiding the use of bracelets. If caregivers were wearing a bracelet, as presented in [7], the skin of the elders would be at risk, because their skin is delicate. Caregivers lift, rotate, move or clean the elders, which require a lot of contact with the elders’ skin, so caregiver bracelets could cause injuries.

#### 4.1.2. Results of Using Accelerometers

Another technology that has shown good results are accelerometers. They are being used like wearable sensors to identify people’s behavior focused on walking, falls and so on. An advantage of accelerometer technology is that it has a tag to link it to a specific artifact and thus we can know the artifact behavior that occurs when the activity is performed. The wide range of recognition of the tags allows recognition of the artifacts in the setting in any moment. We take advantage of this situation to obtain an activity representation. The accelerometer used in this example was a Freescale D3172MMA7456L model. It has a high sensitivity (64 LSB/g at 2 g and 64 LSB/g at 8g in 10-bit mode); a 1-pole low pass filter; the receiver supports 16 sensors per one USB stick allowing identify multiple artifacts, and the power consumption is low voltage operation (2.2 V–3.6 V). We used the default configuration for implementing this technology.

**Figure 7.**
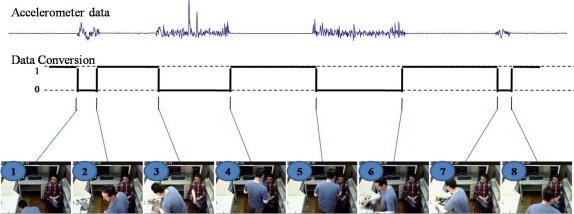
Accelerometer results.

The information produced by the accelerometers is the acceleration measure related to three coordinate axes (*x*,*y*,*z*). Also, it is possible to use the concentrate of these values as the absolute value expressed in terms of g-force (g). Thus, this absolute value was used to show the handling of artifacts as shown in Figure 7 through the values shown with the blue line.

This kind of technology is very sensitive to movements, so the variations start to be recorded when the accelerometer starts to move. An important detail is the broad range of scope that the antenna uses to detect the sensor. The antenna is broad enough to recognize several artifacts that could be used in a setting and because of this technology’s sensitivity, it is necessary to establish a threshold to interpret the input signal. With a threshold, even if the signal is very variable, we can infer that the accelerometer is being used in an activity or event, or otherwise remains immobile. Hence if the input signal of the accelerometer is more than the threshold, a change of state has occurred (from 1 to 0), and when the input signal goes back to the values within the threshold, another change of state occurs (from 0 to 1) as shown in Figure 7.

Figure 7 illustrates several images from the camera installed in the ceiling of the room, in which can be seen each one of the steps when the activity was performed based on artifact handling. Image 1 (in Figure 7) refers to the instant that the artifact is taken for first time. The artifact was held and put on the base location. Image 2 shows when the artifact remains immobile on the base location. Image 3 shows the instant that the caregiver takes again the artifact and moves to the patient, and then the artifact continues moving until it is placed on the patient’s arm as depicted in image 4. At this moment there is a stabilization of sensor values within threshold range and there is no movement. Image 5 shows the instant when the caregiver removes the artifact from the patient and he moves to the base location as shown in image 6. In that moment, the caregiver does a complementary task related to the activity. Finally, the caregiver takes again the artifact and stores it under the base location as shown in image 7. Image 8 refers to the instant when the caregiver goes out to the room. Notice that the beat number is more than the number of beats shown in Figure 2. Therefore, using this kind of technology the number of beats must be increased, because of the sensitivity of the accelerometer.

This kind of technology has been used to get the speed and the orientation of movements of people, specifically to know whether a person is sitting, standing or walking as presented in [17]. Other studies such as reported in [26], presented good results on activity inference through joining two technologies such as RFID and accelerometers. However, in [26] the authors used a bracelet, and as we pointed out in the previous section, bracelets are not appropriate with ERM.

#### 4.1.3. Results of Using Video Cameras

A third technology that has showed good results are video cameras, which produce a continuous video signal. It is a continuous signal of images that shows the setting desired, so that when an image is analyzed, it is possible, in an implicit way, to identify people, their position in the setting, determine whether a person is sitting, standing or resting, and we might even infer the activity.

In order to obtain artifact recognition in a video sequence, it is necessary to develop a series of mathematics or statistical methods that can give as results comprehensible information. Our work uses a statistical method called composite correlation filter. Thanks to this method, it is not necessary to perform image segmentation for artifact recognition, and it is possible to locate multiple artifacts.

Currently, correlation methods have been used because it has been shown that nonlinear filters have tolerance to some object deformations and good performance in the presence of different types of noise [27,28].

Artifact recognition based on correlation methods computes a level of similarity between two images: (i) the reference images and (ii) the test image or the captured setting frame. The image of the workplace scene is used to test the filter in real time and it is matched with a reference image previously recorded and used to train the filter. As a result of correlation filters, we obtained one high value that represents *x* and *y* coordinates, in which the artifact was located in the test image. To identify the artifact using correlation values, it is necessary to establish a threshold that allows us to discriminate those values that are not desired, for instance other artifacts different from the reference images. A filter is associated to each artifact to recognize. In this example, we used vision-based tags recognition (see Figure 5(c3)) but it is possible to use feature-based artifact recognition.

**Figure 8.**
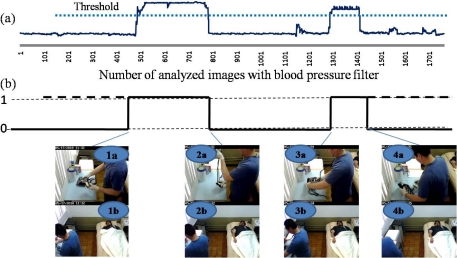
Images correlation results using blood pressure filter (**a**) Correlation values obtained from analyzes a video sequence, raw data; (**b**) Data conversion.

The procedure followed using correlation method is described below: A video camera was installed close to the base location. The camera used was a wvc53gca Linksys model. The video was captured using MPGe-4 format having a resolution from 320 × 240 pixels, the frame rate was 2.4 per second. Storage and processing were done in a model T3500 Dell Precision workstation. Only images obtained by this camera were processed. For each artifact to be recognized on the base location, we built a recognition filter by artifact and a threshold was established (dotted line) as shown in Figure 8(a). This figure represents the results from the correlation between the blood pressure filter (for artifact recognition) and each image of the video sequence. Hence, whether the correlation value is greater than or equal to the threshold, it is accepted as the artifact is being recognized; this moment is designated as correlation value 1. Below the threshold, the correlation value is 0. When a change of state is detected, the current frame is associated with an index and recorded in the database as a beat.

Figure 8 shows several images obtained by the camera installed in the ceiling of the room (1b, 2b, 3b and 4b).The images obtained by the camera installed close to the base location are illustrated (1a3, 2a, 3a and 4a), where can be seen each one of the steps related to the activity performed. These images were recovery from the video thanks to the assigned index. Image 1a and 1b show the instant in which the artifact is placed on the base location. Images 2a and 2b present the time when the artifact is removed from the base location with the purpose of performing the activity. Image 3a and 3b present the instant in which the caregiver again puts the artifact on the base location and the filter recognizes the artifact. Finally, images 4a and 4b shows the instant in which the caregiver removes the artifact and it is not recognized by the filter.

The use of Computer Vision techniques is essential for an AmI system: it gives the possibility to monitor an environment and report on visual information, which is commonly the most straightforward and human-like way of describing an event, a person, an object, interactions and actions of achieving robustness the scene [29], as we presented in this approach. However, artifact recognition through correlation filters has a few disadvantage related to the occlusion or the difficulties to recognize small artifacts. Therefore, we suggested using additional technologies related to artifacts as discussed above, so that they can help to overcome these kinds of drawbacks.

Nonetheless this vision technique is still a good approach for activity recognition because it allows information recovery of several scenarios. This is especially pertinent where caregivers do not wear devices and allows them to perform direct interactions with the ERM where the elders’ skin is very delicate.

At this point three technologies were described, and the objective is to show the similarity between the three activity representation and the RB representation produced by each technology, see Figures 6–8. Each image showed in the referred figures was acquired by each beat produced by the artifact recognition module, and it was possible to link each beat with the equivalent indexed frame of the video sequence captured (by ceiling room camera). So, it is possible to involve several sensors for recognizing places, people, artifacts, and all this information could improve the activity inference process.

#### 4.1.4. Mixing Two Technologies

The manner in which two technologies create and present their information is equal to providing changes of state when the artifacts are being handled. It allows for incorporating their information in a representation, as presented in our approach. After that, we can analyze and represent the results obtained by the artifact recognition that involves the use of several technologies which allows complementing the information related to the setting.

**Figure 9.**
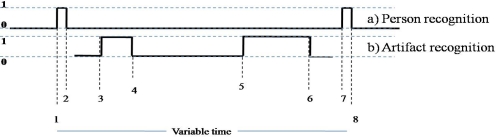
Two technologies used (**a**) Person recognition using RFID technology; (**b**) Artifact recognition through image processing.

In order to observe how the data acquisition obtained by different kinds of technologies can be complemented, we present Figure 9 which shows an activity being performed using two technologies, RFID for people recognition and video cameras for artifact recognition.

The scenario used was configured as follows: a room contained an elder with restricted mobility living there. First, an RFID antenna was installed in the door of the room, and it verifies the input and output when the caregivers enter or leave the room. Two cameras were also installed in the room, one was installed in the ceiling of the room as shown in Figure 5(d) and another was installed close to the base location as depicted in Figure 5(c1) with the purpose of performing the artifact recognition through correlation filters. Figure 9 illustrates the behaviors obtained of the RFID and video cameras technologies. Figure 9(a) shows the person recognition using RFID technology, as this behavior represents the person from the moment he enters the room until he leaves. Figure 9(b) is the behavior obtained from the artifact recognition using correlation filters when the caregiver handled the artifact.

An important detail in this kind of representation is that each change of state is a time stamp. Therefore, this scenario produces 8 time stamps related to particular events using two kinds of technologies as shown in Figure 9.

The strategy of installing a camera in the ceiling of the room and data acquisition of several technologies has allowed us to show information related to the setting and the activity in a visual fashion. Notice that no processing is made to the images obtained by the camera installed in the ceiling of the room, as it just provides indexes of the video sequence recovered once a change of state is produced. Each change of state is related to an image of the video sequence. Hence, the roaming beat (RB) is linked to a set of images in a time span related to an activity.

The RB presented in this subsection shows the interactions when the artifacts are handled through three kinds of technologies: RFID, accelerometer and video camera. The video sequence recording of the entire scene allows us to transform the RBs in a visual representation of the activities. These activities were based on the artifact behavior and joined the information through several technologies and thus complement the information related to the environment. Moreover, it is possible to provide a meaning derived from the behavior produced when the artifact is handled and recognized, as well as the behavior of the people recognition independently which is the kind of input technology. In total, RB allows us to develop an AmI system in a nursing home. This system makes it feasible to integrate multiple sensors with the purpose of modeling artifact behavior and activity interpretation in a healthcare environment focused on elders with restricted mobility.

### 4.2. Recovering Information through Roaming Beat

Based on the results produced by the three technologies, it is necessary to know what kind of information can be derived for the user through visual representation of the RB presented in this study. RB represents a set of images in a time span, and it allows us to recover several kinds of context of the setting such as identity, location, time and activity such as shown by Dey and Abowd [30].

Identity is recovered by artifact/people recognition, depending on the tag used for recognition. Location is recovered by the behavior produced by the artifact. The behavior in our approach is identified through the changes of state of the artifact, where 1 is when the artifact is recognized on the base location and 0 is roaming in the setting when the activity is being performed. The time is recovered by each beat produced so that the first change of state is linked to the date and hour in which artifact recognition was detected. Finally, the activity is the set of RBs produced by one or several artifacts related to a specific activity. Namely, the activity is inferred until that the behaviors of artifacts handled are recognized.

**Figure 10.**
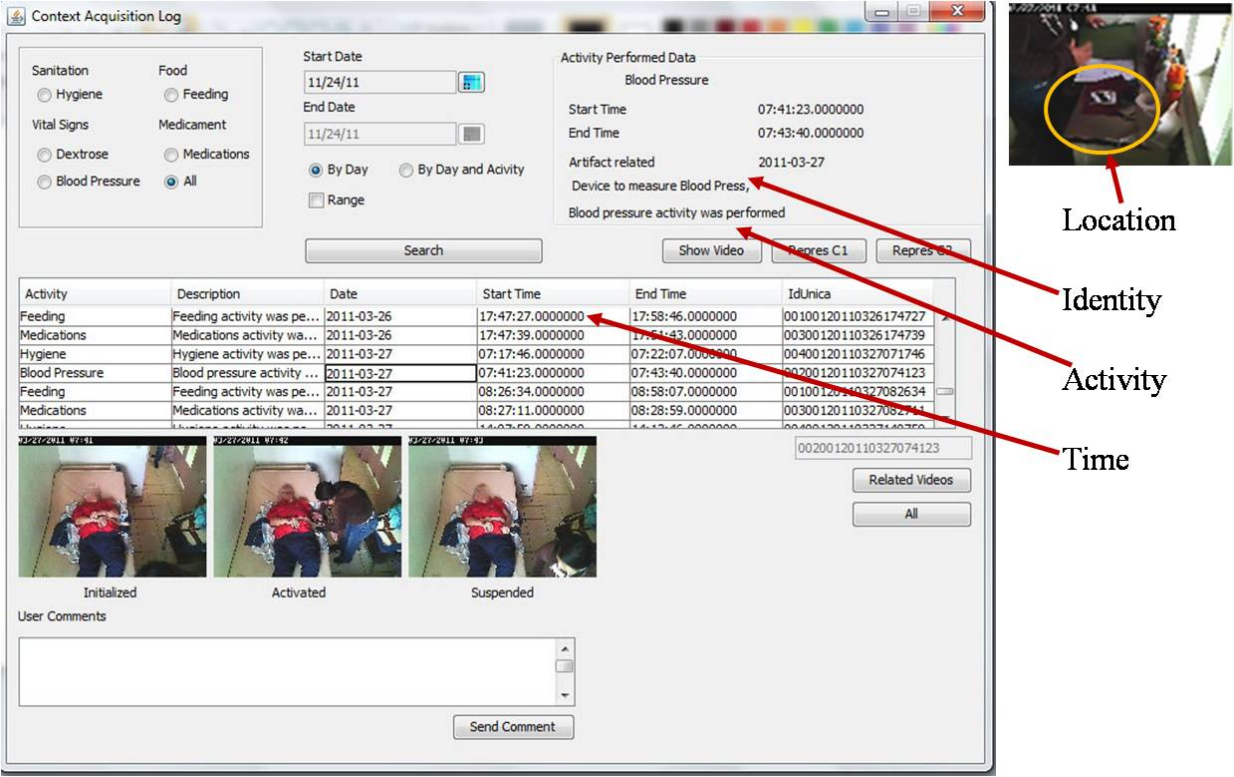
Recovering context through roaming beats.

The information related to the kind of context aforementioned is stored in a database; it creates a log-file related to several events that might be useful for the user as illustrated in Figure 10. Creating opportunities to recover and visualize the information, as well as taking into account the user profiles to show the information, allow taking decisions in a timely manner.

#### 4.2.1. Examples of Information Retrieval

As an example of information retrieval, image 1b within in Figure 8 shows the first beat produced by the device to measure blood pressure. In this image it is possible to identify the person that performed the activity in an implicit way. In the same manner, by observing the image it is possible to know the place where the activity was performed and infer whether the person is sitting, standing or resting. Moreover, the change of state is a time stamp in which an interesting event happened. Hence, each beat represents a piece of information that is used to show the actions that the person performed.

Therefore, the RB can be used for activity representation in a visual manner, showing images of the setting related to the activities performed at specific points of time. These points allow us to create video segments related to the activities, thus it is possible to reduce the query time of videos that are produced by the visual surveillance system, as well as provide contextual information, creating signals of the activity on each beat. Each video segment allows the user to view what happened and determine whether the caregiver’s performance was correct. Further, the user could take appropriate actions to fix the mistakes of the caregivers. Finally, we suggest using image processing as a base to recover information and complement it using other technologies that could represent their information with a RB. It would increase the understanding and meaning of the visualization in specific points of the scene.

**Figure 11.**
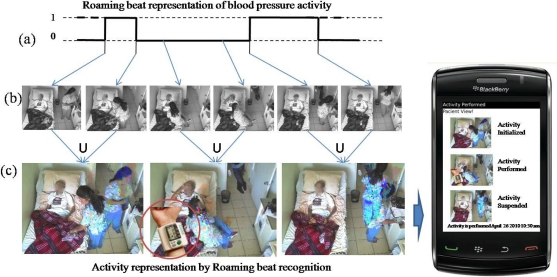
Sending roaming beat representation to a mobile device (**a**) Roaming beat representation of blood pressure activity; (**b**) Images acquired using indexes of a video sequence; (**c**) Joining two images, additional processing.

Another example from our case study involves observing when some activities need to be scheduled and performed in specific times. These activity schedules allow sending notification related to activity omission and performance, depending on the user’s necessities. An example of the recovery of the information based on beats is shown in Figure 11, in which each image is recovered using the index linked to each beat. An additional two images are included that were recovered from two different points when the activity was performed on the patient. These images were joined and sent as a notification through a mobile device.

Finally, the example in Section 4.1.4 shows where two technologies are used and both produce the RB representation. First, when a person is recognized, it is possible to load the preferences based on his profile and show the information in a public display. Once the user preferences are loaded, the AmI system should recognize what actions the user/caregiver wants performed, such as artifact recognition, then show the information (log-file of the artifact or context history) related to the artifact in an automatic way. For instance, the AmI system could present a graph related to the behavior of the blood pressure activity showing the last month’s results. It is even possible to give a recommendation for taking a decision in case the blood pressure results previously were lower.

In summary, the system recognizes a person, an artifact and its behavior; it also records the time related to each beat produced by people recognition and artifact handling. It leaves evidence of the caregiver’s interactions with the patient, as the artifacts were used when the activity was performed.

Up to this point, three examples were presented that showed the opportunities for information recovery through RB using people/artifact recognition. The information was recovered with the purpose of realizing the acquisition of context in a scenario. The context obtained was identity, location, time and activity. These kinds of context were stored and used as context history as proposed in [31].

### 4.3. Practical Case Results (*In Situ*)

As a final step of the practical case, we developed a system based on the framework shown in Figure 1 with the purpose of evaluating the criteria (stated in Section 3.2.) of activity inference in a real scenario. The system is called Context Acquisition Log (CALog) as shown in Figure 10. The evaluation of the system was conducted in a private nursing home for a period of ten days during 12 hours per day. Two video cameras were installed in a room where an elderly patient with restricted mobility (ERM) was living. One camera was installed in the ceiling of the room and another was installed close to the base location (2 meters approximately). The camera specifications were mentioned in Section 4.1.3.

The system was configured for inferring four activities as follows: feeding, blood pressure, hygiene and medications activities. All artifacts were recognized using a vision technique through composite correlation filters and the Table 1 shows the criteria and the relationship between artifacts and activities.

The four criteria by artifact were encapsulated in a process, allowing activity inference in an interleaved or concurrent way. The system was configured in such a way that each process gets an index of the video sequence. It is performed each time that an artifact produces a change of state, and it is stored in a data base.

#### 4.3.1. Activity Inference Results

As a result of activity inference, the system was able to record 81 activities of which 74 were inferred in correct form. These 74 activities followed and accomplished the four criteria shown in Table 1. Table 2 shows the details related to these 74 activities and includes the beats produced by each artifact. The rate of effectiveness for activity inference was 91.35% (*i.e.*, 74 of 81 activities correct).

An important aspect of the hygiene activity is that it does not need all the three artifacts related to it (*i.e.*, paper towel, solution and cream) to be handled in the estimated time span because this activity is not restricted (see Section 3.2 flexible case). As shown in Table 2, the hygiene activity was inferred 21 times, of which 14 were performed using two artifacts and seven were performed using three artifacts. The number of changes of state produced by the paper towel is greater than the other two artifacts because the paper towel in this environment is used for cleaning in several ways within the activity. Also in Table 1 can be seen the paper towel configuration where it needs at least six changes of state to link it with the activity. However, at least two artifacts must follow the established criteria to infer the hygiene activity.

Each artifact behavior was processed independently allowing us to infer the activities in interleaved way. Interleaving was relevant for inferring the feeding and medications activities. In this kind of healthcare environment, medications are supplied to the patients when the feeding activity is performed; in this case, they were supplied at breakfast and dinner. The feeding tray was the first artifact to be placed on the base location and afterward the caregiver took the pillbox located under the base location. The medications activity had a short duration so it was inferred before the feeding activity has finished.

Table 1 shows the criteria configuration for the feeding activity where the defined number of changes of state equal to 2, because the tray remains in the base location until the activity finishes, so this artifact has two changes of state, one when it is placed and another when it is removed. During the time when the tray remains immobile, the caregiver moves from the base location to the patient’s place in several times, taking a distinct artifact (such as plate, cup, glass, *etc.*) from the tray. The artifact configuration of medication activity was defined (≥2) because sometimes the behavior was similar to the tray (artifact related to feeding activity) and other times the number of changes of states was increased two additional changes of states) when the feeding activity was performed

Finally within the evaluation system, the blood pressure activity was always performed in an independent way. There were no other activities started at the same time or in an interleaved way with blood pressure activity. The number of change of states established for this activity is ≥3 because sometimes the artifact remained on the base location once the activity had been concluded. This is the way that the maximum time of waiting criterion, know as quantum (last criterion in Section 3.2), is verified when the last change of state is produced by the artifact.

## 5. Discussion

Integration of multiple sensors in healthcare environments produces a huge amount of information related to the setting. The challenge is taking advantage of the information produced to address the user’s needs, so that at the moment when the information is obtained, there are context signals applicable to the user, as AmI has established. Therefore, our proposal is focused on generalizing an interpretation of information for activity inference based on handling artifacts. Using several input sources it is possible to give a signal with the aim of providing capacity to the computer to view the world around us and in this manner to create applications that allows for information recovery in an easier way.

In the literature there are a lot of approaches where ontologies, frameworks and other design insights are shown to develop tools for activity inference. Usually, before developing a system, developers and designers take into account these tools, but as a consequence, they may make prejudgments caused by lack of knowledge related to the scenario and produce a bad design for developing systems [32,33].

To solve these drawbacks and get more information related to the scenario, it is necessary to be involved into the scenarios as is proposed in this work through activity characterization focused on the desired scenario.

This characterization allows us to know the role of the artifact in use in this particular environment and it helps us to define our concept called roaming beat (RB). The concept, based on the role of the artifact in use, is an important part of the activity inference, as is shown by activity theory [23]. In the same way, our activity inference approach shows features in which activity theory elements are involved, such as when the user is handling artifacts and the role that artifacts play to accomplish a goal (e.g., activity). These considerations allow us to overcome some challenges in artifact recognition such as interleaved and concurrent activities.

Entire analysis of the scenario gave us important results of usefulness from information based on real data; it creates the opportunity to search for appropriate ways to send information related to the environment in an automatic and smart manner. Some research has shown the need for monitoring the healthcare environment, where the monitoring is focused on the caregivers to give a whole awareness of the caregiver over risk situations [34]. Therefore, our framework is fitted to fulfill this need.

**Figure 12.**
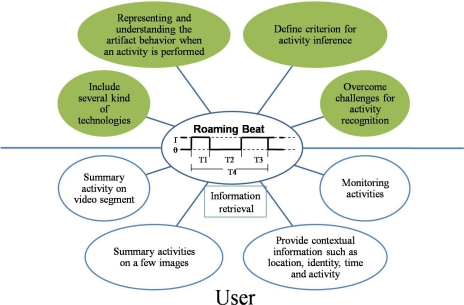
Roaming beat vision focused on designers, developers and user.

An important aspect is the fusion of the information that is possible to obtain from several sources, and thus provide in automatic, smart and timely manner appropriate information based on the environment. In this paper we contribute our activity inference approach and generalize it for interpretation of activity inference. We believe that by using our RB concept it is possible to provide an understanding of activities. Our framework is useful for designers and developers to accomplish the challenges of activity inference for context acquisition in a scenario. Also they can recover the information in an easier manner and thus can develop tools focused on the user, as depicted in Figure 12.

## 6. Conclusions

In this paper we presented the framework called SCAN that is based on the use of a concept named roaming beat (RB) for activity inference. The framework is divided in three modules: In the first module, data acquisition is obtained by three technologies; in the second module, the activity inference is obtained based on handling artifact; and finally the third module is used for activity representation. We showed that with this framework is possible to involve three kinds of technologies and also to obtain a similar interpretation related to handling artifacts when an activity is performed. We believe that this framework can be used in other kinds of settings; *i.e.*, where there exists a base location, there is the handling of artifacts, and the setting can be recorded using video cameras. We presented the framework description, the RB definition and its analysis, and the steps to obtain the activity inference.

The understanding of the setting was based on a qualitative analysis. We suggest using the grounded theory to obtain features related to artifacts’ behavior. By using grounded theory, it is possible to identify many details in the setting, and based on these details it is possible to implement AmI systems. The analysis conducted in the case study allowed us to identify six artifacts involved in four activities which are feasible to implement through artifact recognition.

Each artifact creates its own RB. Each RB is recognized by handling the artifact on a base location. Artifact recognition is obtained by three kinds of technologies: RFID, accelerometer and video cameras. The latter was used with composite correlation filters. Each RB is recorded in a database and it is used to create video and annotation indexes in an automatic way. Using these indexes we were able to recover the processed images. Also, these indexes are linked to video sequences related to other cameras installed in the setting. These images are supplementary context information of the setting and using this information it was possible to create an activity representation based on each RB. Several images were arranged with the purpose to give a representation related to the activity. It can be helpful to get a better context representation of the activities.

Framework description, roaming beat description and CALog system allowed us to tackle distinct problems such as the feasibility of implementing AmI systems, also to show the feasibility for accomplishing the challenges related to activity recognition based on artifact recognition. In this way, we showed the applicability of our framework in an AmI system.

We showed a practical example in a nursing home. In this scenario, the process of care is performed by caregivers who give a better quality of life to elders. Additionally, we presented the lab results implementing three technologies. We performed the blood pressure activity and it was possible to obtain a similar analysis of the activity in the data acquisition from the three technologies. Also, we presented a scenario where two technologies are involved to show how the RBs are obtained. This example allowed us to show the integration of three technologies applied for activity inference.

Two examples were presented which showed how it is possible to recover an activity representation. It was also shown how it is possible to apply image processing and prepare the information for sending it to mobile devices. In this manner, the system has the possibility of summarizing the activity performed in few images or a short segment of video.

Finally, we presented the implementation of our system *in situ* that includes the RB concept and SCAN framework. The system recognized 81 events where 74 activities are inferred. The activity inference criteria were accomplished by the artifacts’ behavior. Our system displayed an activity inference effectiveness of 91.35%.

Our work suggests that the activity representation presented in this work can be an alternative tool to summarize and present an activity in a short video. A limitation of this approach is that if there is no artifact recognition on the base location, then activity inference is not obtained. Nevertheless, our intention is to extend our SCAN framework to other situations or contexts that could make use of a base location format, such as in hospitals, home or commercial kitchens or in other environments with similar features.

As our further research, we know that this work will solve several problems that our informant mentioned us in the case study, such as activity visual evidence for caregivers’ activity coordination when a shift rotation occurs, for detecting activity omission and abuse in ERM. The information will even be used as learning method for caregivers.

Since our approach has been tested in a private nursing home, the staff there is interested not only on monitoring ERM but also on independent elderly persons. This implies the recognition of other activities that perform the caregivers. Recognizing additional activities increase the cost, but it helps to infer activities on an automatic way to improve the quality of life for elderly people (independent or dependent). Therefore, another research line to follow is the study of the cost-benefit of doing automatic inference of activities against traditional operation of a nursing home.

Finally, we must be aware of the video stream produced by cameras because it involves the elderly, staff and relatives, but their perceptions have not been considered in this research. In future, we can find out their perceptions as opportunities for improving our concept and creating other kinds of tools focused on the user. Privacy and security of data are interesting topics that would be considered as future work.

## Figures and Tables

**Table 1.** Criteria by artifact with its related activity.

		**Activities**					

		**Feeding**	**Blood pressure**	**Hygiene**			**Medications**

**Features**	**Artifacts**	Tray	Device to measure blood pressure	Physiological solution	Body lotion/cream	Paper towel	Pillbox

	**ΔT (minutes)**	5–50	5–10	10–30			3–5
	**# beats established**	2	3–4	≥3	≥3	≥6	≥2
	**Quantum (min)**	5	5	10	10	10	3

**Table 2.** Activities inferred and the number of beats.

**Activities**	**Inferred**	**Number of Beats**
Hygiene	21	279
1. Paper Towel (PT)		170
2. Solution (Sol)		91
3. Cream (Cre)		18
Blood Pressure	14	56
Feeding	23	52
Medications	16	52
**Total**	**74**	**439**
